# Petroclival meningiomas: radiological features essential for surgeons

**DOI:** 10.3332/ecancer.2019.907

**Published:** 2019-03-05

**Authors:** Luca Nicosia, Salvatore Di Pietro, Michele Catapano, Gaia Spadarella, Lara Sammut, Christine Cannataci, Federico Resta, Paolo Reganati

**Affiliations:** 1 Breast Radiology Department, European Institute of Oncology, 2014, Via G Ripamonti 435, Milano, Italy; 2 Medical Imaging Department, Mater Dei Hospital, Triq Dun Karm, MSD 2090 Msida, Malta; 3 Neuroradiology Unit, San Giuseppe Hospital, Milano Via San Vittore 12, 20123 Milano, Italy; * Luca Nicosia and Salvatore Di Pietro contributed equally and share first-authorship

**Keywords:** petrocliveal meningioma, neurosurgery, neuroradiology

## Abstract

Petroclival meningiomas (PCMs) have always been a challenge for surgeons because of their difficult anatomical location. The role of radiology in providing precise indications regarding the tumour site and aggressiveness plays a major part in guiding the subsequent therapeutic process. The purpose of this review is to provide a set of the main radiological features helpful in the management of PCMs towards the most correct therapeutic approach.

We aim to offer a radiological overview to allow the patient to be directed to surgery with the least possible risk of complications.

## Introduction

Meningiomas are meningothelial cell neoplasms, which typically attach to the inner surface of the dura matter. They are a very frequent intracranial neoplasia, routinely diagnosed in clinical practice, representing approximately between 16% and 20% of overall endocranial tumours [1].

According to the WHO classification of central nervous system tumours published in 2016 [2], most meningiomas are benign and categorised as WHO grade I. Only a few are associated with less favourable clinical outcomes and categorised as WHO grade II or III, indicating malignancy.

The most common histology is represented by meningothelial meningioma, followed by fibrous and transitional subtypes: as a whole, they account for 80% of meningiomas [3]. Meningiomas usually arise from the arachnoidal cap cells of the leptomeninges; however, they can be located in almost any area of the brain, even in the ventricular space [4]. Regarding this, interestingly, many studies have tried to show how some atypical histologies correlate more with unusual sites [5]. Among these, petroclival meningioma (PCM) can represent an astonishing example; it only accounts for 11.42% of all posterior fossa meningiomas and deserves a very careful evaluation [3, 6].

**Figure 1.**
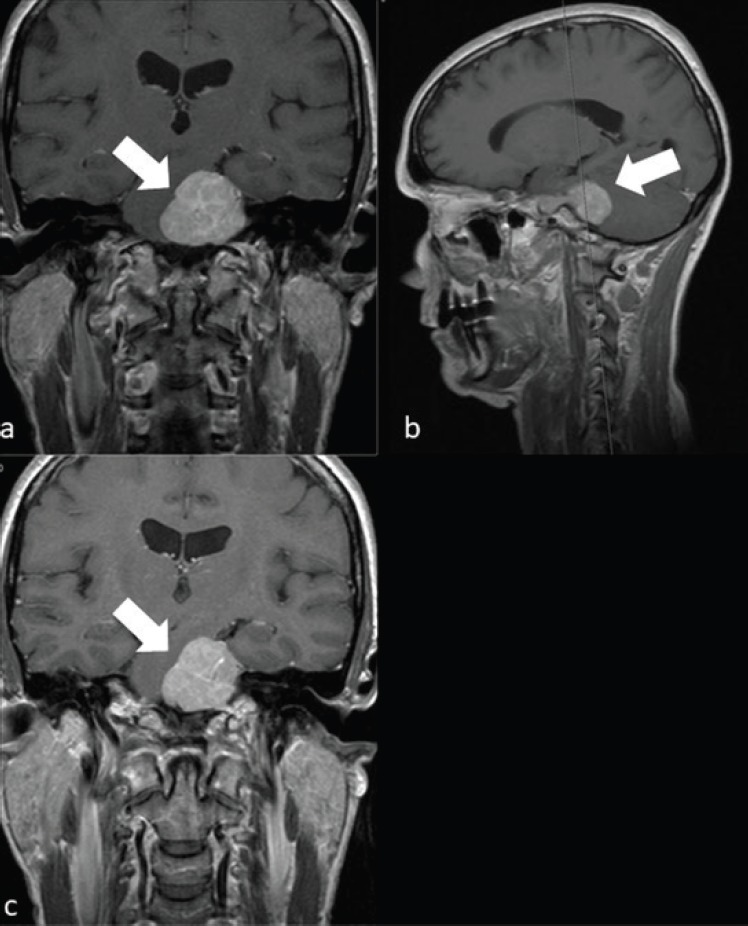
A 62-year-old man with a left PCM. a) Left PCM on coronal T1 sequence. b) Left PCM on T1 sagittal sequence. c) Left PCM on T2 coronal sequence.

This kind of neoplasia arises generally from the upper two-thirds of the clivus, intimately associated with the brainstem, basilar artery, perforating arteries, and multiple cranial nerves and is partially obscured by the temporal bone. This particular anatomical site makes frequent surgical problems easy to understand [7], as shown in Figure 1.

**Figure 2.**
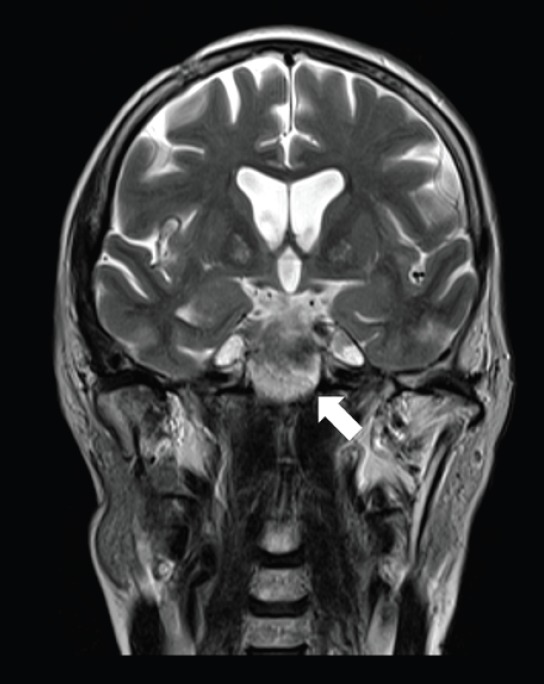
A 57-year-old man with PCM of midline on coronal T2 sequence.

**Figure 3.**
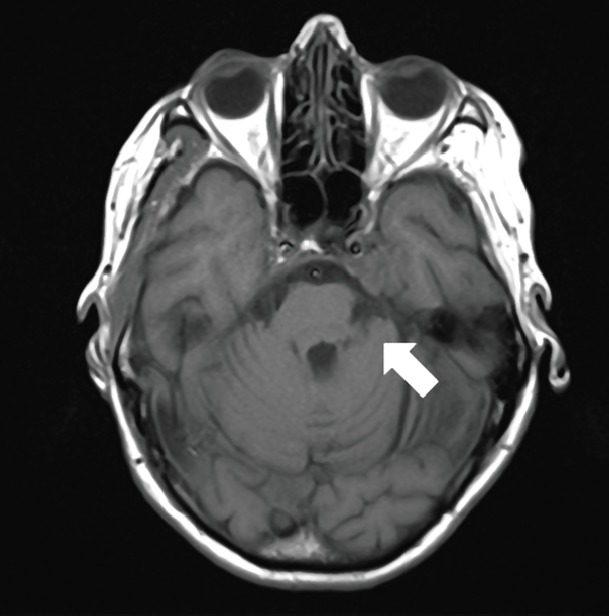
A 74-year-old woman with a PCM of the clivus on left side on axial T1.

Castellano and Ruggiero first understood that posterior fossa meningioma could have different prognostic evolution, thanks to extensive postmortem examinations. They considered five categories: convexity, tentorial, posterior surface of the petrous bone, clivus and foramen magnum [8]. However, the term PCM was not well defined until 1996 when Couldwell *et al* [9] suggested a precise definition excluding lower-third clival-located tumours and lateral petrous or petro-tentorial lesions. In Figures 2 and 3, we can see typical examples of PCMs, it was clear from the beginning that this neoplasia could not benefit from a wait-and-see approach: the PCM seemed to have a relentless progression, needing proper and immediate treatment. Surgical excision of PCM indeed proved to be challenging for surgeons, both in terms of difficulties in accessing the region and of severe postoperative morbidity and mortality [8, 10]. Mortality from resection of PCMs exceeded 50% before 1970 [11], to such an extent that some authors considered these tumours to be largely unresectable until the 1970s [12].

Surgery of PCM can nowadays be performed with excellent results after a presurgical safety assessment, including a detailed analysis of imaging characteristics of magnetic resonance imaging (MRI). The radicability of tumour resection is strongly associated with some anatomical features, easily evaluable with the imaging: tumour size, composition and aggressiveness, adherence to the brainstem, proximity to the neurovascular structures and tumour extension into the cavernous, petrosal sinus. Preoperative magnetic resonance is always necessary in order to predict the difficulties related to surgical resection: until now, the imaging features that might help to predict the complications and the feasibility of surgery of PCMs are not yet well characterised. The purpose of our study is to review the main works in the literature to establish the radiological characteristics that allow the patient to be directed to surgery with the least possible risk of complications [11].

We searched on PubMed for the terms ‘meningioma’, ‘petroclival’, ‘surgery’ and ‘imaging’ in varying combinations. Our analysis included recent papers with the largest numbers of patients, trying to identify the principle features that can be related to a successful surgery. Studies not regarding meningiomas of the petroclival region were excluded.

Mayberg and Symon were the first authors who approached neuroimaging focussing on PCMs: the use of pre-operative computed tomography (CT) scans and cerebral angiography has reduced the overall mortality to 9%. [13]. Sekhar and Schramm reported the use of MRI for preoperative evaluation of PMC for the first time in 1987 [14]. The importance of MRI in the preoperative assessment of these lesions was highlighted in 1996 by Sekhar *et al* [15], especially the great importance given to the invasion of the pial layer; when pial is involved, the subarachnoid plane is lost and this leads to a much higher risk of complications. Pial invasion by the tumour is generally related to the loss of the arachnoidal plane on T1 images and to the presence of blood supply from the vertebrobasilar artery discovered during angiography. Even if the pia-arachnoid plane of the brainstem is partially lost, total dissection from the brainstem is feasible without major neurological deficits when the brainstem perforating arteries have a good flow. Whenever it is not possible to remove an intracranial portion of the meningiomas as a consequence of a tenaciously adherent portion of the tumour, radiosurgery is the best option to treat the residual lesion. In case of the second operation on the surgical site, dense adhesions, which are a consequence of the first intervention, could increase the surgical risk involved during surgery time [16]*.*

The petroclival region is a surgical space circumscribed anteriorly by the clivus, laterally by the petrous apex, medially by the brainstem and posteriorly by the internal acoustic canal. It goes from the dorsum sellae to the foramen jugularis. It is intersected by cranial nerves IV to XI and by the basilar artery with its branches [17].

The most important tips and tricks are represented by estimating the volume of the osseous area of the Kawase space, acquired by CT, reported by Altieri *et al* [18]. This area has a mean volume of 1.89 ± 0.52 cm^3^. This evaluation plays an important role in planning anterior petrosectomy. Although the portion of bone resected is a pyramid (considered a three-dimensional space) so as to prevent mistakes caused by the three-dimensional area, in that study, to obtain a good plan, the images were collected and worked out by Orsix for Mac (Pixmeo Sarl) [19]. In an MRI analysis conducted by Nakano *et al* [20] on 51 patients with meningioma, multiple important factors were found. Factors that correlated with the presence of brain oedema on the univariate analysis were the signal intensity of tumour on T2WI, peritumoural rim, tumour size and shape of tumour margin. Moreover, loss of peritumoural rim and irregular tumour margins were considered as parameters to report cortical penetration of the tumour.

In the literature, there are some important analyses to estimate radiological features capable to give the chance of a satisfactory tumour removal: meningiomas usually appear as round-lobular shaped masses in the extra-axial space with thin and definite margins. Some of the most important features of PCMs are summarised in Table 1. They typically develop with a broad-based dural attachment, often they displace the brain inwards during their expansive growth [21]. Rarely, they may, however, exhibit a more infiltrating growth pattern over the dura, configuring the case of the ‘meningioma en plaque’, most commonly placed along the sphenoid ridge or the convexity [22].

This kind of lesion usually shows isointensity to slight hypointensity relative to grey matter on the T1-weighted sequence; whilst they appear isointense to slightly hyperintense relative to grey matter on the T2 sequence. After contrast administration, meningiomas typically demonstrate rapid, rich and homogeneous enhancement (except for occasional not enhancing components such as areas of central necrosis or calcification). Calcification is a typical finding, best studied on CT. On MRI, calcification is best identified on susceptibility-weighted images as areas of low signal intensity

Contrast is of great help in ‘en plaque meningiomas’ evaluation since they typically appear as asymmetric thickened sheets of enhancing dura [1].

## Assessing structural features of meningiomas and their aggressiveness using imaging

Several studies have been performed assessing the correlation between the MRI findings and the histology of meningiomas. Almost all studies have shown that the signal intensity on T1-weighted images does not correlate with tumour histology or histological subtype; in fact, most meningiomas are isointense to the brain cortex regardless of tumour histology [23–31]. Data regarding the correlation of T2-weighted and proton density (PD) images with tumour histology are controversial. Some series report no statistically significant correlation between the two, [24] whilst other studies show a statistically significant correlation between the two [23, 25, 27, 29, 31, 32]. Elster *et al* [30] showed that all meningiomas which were hyperintense to the brain cortex on T2-weighted images were of the syncytial or angioblastic type; significantly hypointense tumours on T2-weighted imaged were mainly composed of fibrous and transitional areas; whereas isointense tumours included the transitional and syncytial meningiomas. Chen *et al* [28] demonstrated that malignant and angioblastic meningiomas were in most cases hyperintense on T2-weighted imaging, fibroblastic and transitional meningiomas were hypo or isointense to brain cortex on T2-weighted imaging, whilst meningothelial tumours, compared with fibroblastic and transitional meningiomas, appeared more commonly as hyperintense lesions. Zee *et al* [33] showed that T2 hyperintensity was more often depicted in aggressive, angioblastic and meningothelial meningiomas. Suzuki *et al* [26] report that fibroblastic meningiomas demonstrated low signal intensity on T2-weighted images, in keeping with features of a ‘hard’ fibrous tumour, whilst angioblastic meningiomas showed a high signal intensity. Soyama *et al* [34] described 40 surgically treated intracranial meningiomas, whereby the lesion’s signal intensity on T2 was correlated with the histological subtypes. T2 signal intensity scores were assigned. The mean signal intensity scores on T2-weighted imaging of the fibrous type of meningioma were lower than those of the other subtypes. These scores proved to correlate T2 hypointensity with the lesion’s fibrous elements. Maiuri *et al* [24] results were similar to those of Elster *et al* [30] and Chen *et al* [28], supporting the idea that signal intensity of meningiomas on T2-weighted images does correlate with the histological subtypes. Maiuri *et al* [24] evaluated the intraoperative consistency of 35 intracranial meningiomas [19]. All patients perform a preoperative MRI which includes T1 and T2-weighted sequences and gadolinium-enhanced images [21]. With regards to fibroblastic meningiomas, Maurer *et al* [11] demonstrated that all these show hypo or isointensity on both T1 and T2-weighted imaging, with none showing high signal intensity. This is in keeping with the ‘hard’ fibrous nature of the fibroblastic subtype whereby thin cells are stacked in a rich collagenous matrix. Histological subtypes are usually of the fibroblastic or transitional types. Syncytial, angioblastic meningiomas mostly appeared hyperintense on T2-weighted imaging. This would be in keeping with the amount of micro-cystic changes, high cellularity and dilated blood vessels of this subtype on a histological level. The same author showed that high tumour signal intensity on T2-weighted imaging always correlated with a soft tumour consistency and/or a vascular lesion intra-operatively. Meningiomas isointense on T2-weighted imaging were primarily transitional and partly fibroblastic and syncytial. Transitional meningiomas were mainly isointense (60%) or slightly hyperintense (40%). Lipomatous meningiomas showed marked hyperintensity on T1-weighted imaging. As their name implies, they are characterised by the partial or total metaplastic transformation of tumour cells into adipocytes. The transformation into a fatty cell with similar MR signal intensity may also be observed in the xanthomatous type, a very rare variant of the angioblastic meningiomas or, more rarely, of the syncytial and fibroblastic types [24]. This, therefore, shows that the lesion signal intensity on MRI may be an effective preoperative evaluation of intracranial meningiomas not only to pre-empt tumour histology but also the degree of tumour vascularity on MRI; thus, pre-empting intra-operative haemorrhage. Chen *et al* [28] also demonstrated that hyperintensity on T2-weighted images was able to predict microscopical hypervascularity. This is confirmed by the fact that meningiomas of the angioblastic histological subtypes are found to be almost always hyperintense on T2-weighted images [12, 14]. On another note, it has been shown that the degree of calcification of meningiomas does not determine tumour resectibility. With regards to the degree of brainstem compression, this has been shown to not influence clinical outcome. An irregular tumour margin close to the brainstem was reported in 83% of all PCMs that were subtotally removed, whilst an irregular tumour margin close to the brainstem was reported in 33% of totally resected lesions. This observation showed statistical significance.

Good correlation was also demonstrated between tumour resectability and certain preoperative findings such as the presence or absence of an arachnoidal cleavage plane; an arachnoidal cleavage plane was preoperatively shown in 80% of totally resected PCM whilst this was not demonstrated in 88% of the subtotally removed tumours. On the other hand, brainstem oedema did not correlate with incomplete tumour removal. Up till this date, no clear correlation has been demonstrated between tumour consistency and surgical outcome. Also, the values of apparent diffusion coefficient (ADC) in MRI diffusion images seem to have an excellent prognostic value in identifying the behaviour of meningiomas: Hakyemez *et al* [35] reported 39 patients with meningioma, in which the mean ADC value of benign tumours was significant higher than the ADC value of atypical/malignant meningiomas. Other authors also remarked this topic: atypical and malignant meningiomas had lower ADC values compared with benign lesions [36]. Tang *et al* [37] described a statistically significant correlation between ADC and Ki-67 protein in low-grade and high-grade meningiomas.

An ADC cutoff of less than 0.70 × 10−3 mm^2^ s^−1^ seems to be helpful in differentiating aggressive meningiomas from low-grade tumours, whilst an ADC cut-off of greater than 0.85 × 10−3 mm^2^ s^−1^ in identifying low-grade meningiomas [34].

A study of Rempel *et al* [38] underlined the importance of a biomarker: secreted protein acidic and rich in cysteine (SPARC) that consists of a secreted, extracellular matrix (ECM)-associated associated glycoprotein that is involved in the modulation of cell adhesion, migration and angiogenesis during development. It plays two fundamental roles in brain tumour pathobiology: angiogenesis and tumour invasion. Increased SPARC expression is associated with the invasive phenotype in meningiomas, irrespective of tumour histological grade. SPARC is a marker able to identify lesions potentially or actually invasive that are otherwise histomorphologically benign and for which there is no invasive tumour/brain interface available for pathological assessment. Other molecules expression such as Vav3, SPARC, p-Akt, cyclin D1 and Ki-67 were found in meningiomas and correlate with meningioma invasiveness, aggressiveness and recurrence [39]. More sophisticated MRI technologies like fractional anisotropy (FA) and magnetic resonance spectroscopy (MRS) have also been used to analyse meningiomas. Kashimura *et al* [40], by estimation of FA as measured by MRI, found in 29 patients with intracranial meningiomas (11 hard–18 soft) that the calculated FA values of fibroblastic meningiomas were higher than those of meningothelial meningiomas; the FA values of firm tumours were higher than those of soft tumours. In magnetic resonance spectral pattern, there are some peculiar features of both atypical and typical meningiomas such as elevated Cho’s level, presence of Ala and absence or limited amount of creatine and N-acetyl-aspartate and Cr. However, MRS is not able to distinguish typical intracranial meningiomas from atypical meningiomas in preoperative setting. [41]

Reagrding this, there are also novel methods to predict preoperative meningioma grade. Peng *et al*. performed texture and shape analysis to quantitatively evaluate tumour heterogeneity and morphology on 131 pts by three texture features and three shape features and they have found significant differences between high-grade and low-grade meningiomas related to features of texture and shape; so it can be reliable in the preoperative determination of meningioma grade and seems to have potentially effective clinical application [42]. In another study, 175 meningioma patients were applied 15 radiomic (quantitative) and 10 semantic (qualitative) features to estimate the imaging phenotype; 12 radiographic features (eight radiomic and four semantic) were correlated with meningioma grade. The results reported that radiomic and sematic classifiers could obtain a good prediction of meningioma grade with AUCsem = 0.76 and AUCrad = 0.78 [43].

In the new era of precision medicine, radiomics is an emerging translational field of research aiming to find associations between qualitative and quantitative information extracted from clinical images and clinical data, with or without associated gene expression to support evidence-based clinical decision-making. The concept underlying the process is that both morphological and functional clinical images contain qualitative and quantitative information, which may reflect the underlying pathophysiology of a tissue. Radiomics’ analyses can be performed in tumour regions, metastatic lesions, as well as in normal tissues. The radiomics quantitative features can be calculated by dedicated software, which accepts the medical images as an input. Despite many tools developed for this specific task being user-friendly in terms of use and well performing in terms of calculation time, it is still challenging to carefully check the quality of the input data and to select the optimal parameters to guarantee a reliable and robust output. The quality of features extracted, their association with clinical data, and also the model derived from them, can be affected by the type of image acquisition, postprocessing and segmentation. This article summarises the major issues regarding this multistep process, including radiomics, CT and MRI [44].

## Conclusion

The radiological features of PCMs are of great importance for clinicians in the choice of the most appropriate therapeutic approach.

Where surgery is possible (considering the consistency, aggressiveness and invasion of the surrounding structures by the tumour), radiological images play an important role in order to choose the best surgical approach.

The profound knowledge of the radiological characteristics of the PCM is of great importance to assure the best management of patients with this type of lesion.

Many new and innovative methods are being developed over recent years to analyse the radiological features of a meningioma, offering opportunities with great potential and interest.

## Disclosure statement

Nothing to declare.

## Funding

No funding was received for this article.

## Conflicts of interest

All authors declare no conflict of interest.

## Figures and Tables

**Table 1.** Papers that describe the main features of PCMs (12).

Author	Year	Importance of the study
Castellano and Ruggiero [8]	1953	They considered that posterior fossa meningioma could have different prognostic evolution.
Couldwell *et al* [9]	1996	They suggested a precise definition of PCM, excluding lower-third clival-located tumours and lateral petrous or petro-tentorial lesions.
Sekhar *et al* [15]	1987	They reported the use of MRI for preoperative evaluation of PMC for the first time
Sekhar *et al* [15]	1996	They reported that when pial is involved, the subarachnoid plane is lost and it means a much higher risk of complications.
Nakano *et al* [20]	2002	They reported a correlation on the univariate analysis between the presence of brain oedema and the signal intensity of tumour on T2WI, the peritumoural rim, as well as the tumour size and shape of tumour margin.
Elster *et al* [30]	1989	They showed that all meningiomas which were hyperintense to the brain cortex on T2-weighted images were more aggressive.
Zee *et al* [33]	1992	They showed that T2 hyperintensity was more often depicted in aggressive, angioblastic and meningothelial meningiomas.
Suzuki *et al* [26]	1994	Showed that angioblastic meningiomas showed a high signal intensity on T2.
